# Reporting research antibody use: how to increase experimental reproducibility

**DOI:** 10.12688/f1000research.2-153.v2

**Published:** 2013-08-23

**Authors:** Matthew A Helsby, Joe R Fenn, Andrew D Chalmers

**Affiliations:** 1 Department of Biology and Biochemistry, University of Bath, Bath, BA2 7AY, UK; 2 CiteAb, Bath, BA1 1UD, UK

## Abstract

Research antibodies are used in a wide range of bioscience disciplines, yet it is common to hear dissatisfaction amongst researchers with respect to their quality. Although blame is often attributed to the manufacturers, scientists are not doing all they can to help themselves. One example of this is in the reporting of research antibody use. Publications routinely lack key details, including the host species, code number and even the company who supplied the antibody. Authors also fail to demonstrate that validation of the antibodies has taken place. These omissions make it harder for reviewers to establish the likely reliability of the results and for researchers to reproduce the experiments. The scale of this problem, combined with high profile concerns about experimental reproducibility, has caused the Nature Publishing Group to include a section on antibody information in their recent Reporting Checklist for Life Science Articles. In this commentary we consider the issue of reporting research antibody use and ask what details authors should be including in their publications to improve experimental reproducibility.

## Antibody information is routinely omitted from publications

Neuroscience, cancer research, regenerative medicine, infection and immunity, cell biology and cardiovascular research are just some of the fields in which research antibodies are commonly used. The sheer scale of their use is illustrated by huge sales, estimated to be worth in excess of $1.6 billion annually ^1^. Despite, or perhaps because of this widespread use, it is common to hear dissatisfaction among research scientists about the quality of these antibodies ^2– 4^. The finger of blame is often pointed at the manufacturers, yet it is questionable whether scientists themselves are doing everything they can to help the situation; surely not all problems can be placed at the door of the antibody manufacturer. One example of scientists not helping themselves is in their reporting of antibody use. There are many cases of good practice (For example ^5^) and detailed reporting, but all too frequently authors omit key details. These include the host species and code numbers, but even the source of the antibody may be left out. This makes it harder for reviewers to establish how well characterised the antibodies are and thus how reliable the data presented are likely to be. It also makes it more difficult for other researchers to accurately reproduce experiments.

Failure to report key information is not a new problem ^2, 6^, but recent developments have increased efforts to find a solution. In particular, experimental reproducibility has been thrust into the limelight by high profile cases. For example, a study of "landmark" cancer research papers found that scientific findings from only 11% of them could be repeated ^7^. Taken at face value this is a shocking statistic and, in an attempt to try to improve experimental reproducibility, the Nature Publishing Group have recently introduced a reporting checklist for life science articles ^8^. This checklist highlights research antibodies as a reagent type for which reporting could be improved. A key question is; what information to provide? In this commentary we consider what information authors should be including in their publications to help improve experimental reproducibility.

## Key details for reporting antibody experiments

Publications need to report core information regarding the antibodies that were used. This should include the name of the antibody, the company/academic who supplied the antibody, the host species in which the antibody was raised and whether the antibody is monoclonal or polyclonal. In addition, the catalogue or clone number needs to be mentioned. The catalogue or clone number is commonly omitted from current publications, but is important as large antibody companies will often have multiple antibodies to the same target, a unique identifier is therefore essential to allow unambiguous identification of the antibody concerned. For this reason the first step in improving reporting should be to make it mandatory for authors to include core antibody information, including a code or clone number for the antibodies they use.

A second type of information that should be reported relates to experimental details. The application the antibody was used for is of central importance. This information is normally present, but it can be hard to extract if the antibody information is listed in a ‘Materials’ section and separated from descriptions of the techniques. Having the antibody data and application data closely linked would avoid potential confusion. Furthermore, if a study uses samples from more than one species then it is also important to clearly link which antibodies were used in which species.

There are other features that could also be reported which may be particularly relevant to certain studies. For example, the antibody batch number is rarely included in methods sections, but it is common to hear concern about variability between different antibody batches. This is often anecdotal, but there are some published examples ^9, 10^. This type of variability is likely to be a particular issue with polyclonal antibodies ^2^, but may affect monoclonal antibodies ^11^. We encourage scientists to report cases in which variability has been found and in these examples include batch numbers. Reporting the final antibody concentration or dilution is another piece of information which can help other researchers, especially if optimisation was required during the study.

It has been proposed that scientists should know the antigen which was used to raise the antibody ^3^. There are exceptions, for example where antibodies have been raised from a cell/tissue lysate and the antigen is unknown, but for most cases the antigen or at least its location within the protein should be known, as it may have implications for interpretation of the results. In cases where it is relevant to the study authors should be encouraged to report the antigen location. Finally, there will be details of particular importance for individual techniques, we focus on research antibodies, but studies reporting therapeutic use would be an example in which specific details such as purity and dose need to be reported.

## Antibody validation

The Nature Publishing Group checklist, mentioned above, requires authors to demonstrate that every antibody used in their study has been validated for use in each of the specific experiments and species used. The experimental process of antibody validation is complex, with the most rigorous methods being comparison of wildtype vs a knockdown/knockout tissue and/or use of a second antibody to a different epitope. The validation must also be carried out for each experimental setup as specificity in one application, or even fixative, does not mean an antibody will be specific in another. It is also the case that the details that should be reported to demonstrate validation will be different for each application. For more information on antibody validation we highly recommend the following publications ^12– 18^.

Our focus is on how to report antibody validation, which can be achieved in a number of ways. If an antibody has not been previously validated for the specific combination of application and species used, then it should be mandatory that validation be carried out and reported. This can often be included as supplementary information.

If the antibody has previously been validated then one or more citations could be given to highlight the validation. Alternatively, the publication could reference the antibody validation profile from publicly available databases such as 1degreebio, Antibodypedia, CiteAb or pAbmAbs (A more extensive list of databases is available from Pivotal Scientific). Again it is important that antibody suppliers and codes are used in publications so that each antibody can be unambiguously identified and the degree of previous characterisation assessed. If new validation has been carried out then this could also be deposited in a public database and the database cited, instead of or in addition to putting the data in the supplementary information. Including information to show validation has occurred would help reviewers and other researchers accurately assess the results.

## Change will require help from journals and reviewers

It seems likely that significant change is not going to occur unless journals take a lead and encourage it by adding antibody reporting guidelines to their instructions to authors. The success of this has been demonstrated by the Journal of Comparative Neurology which has had extensive guidelines in place since 2006 and the Journal of Visualised Experiments which requires a table of materials, including catalogue numbers for all the reagents used, to be reported. The Nature Publishing Group checklist should improve reporting in their journals and it is encouraging to see that following publication of version 1 of this manuscript F1000Research and PeerJ have added our proposed guidelines to their instructions for authors (described below). Once a journal has added guidelines it will be crucial that peer-reviewers are encouraged to evaluate the reporting and enforce the guidelines.

## A simple format for reporting antibody information

Based on the points discussed above we would suggest that journals adopt, and researchers use, the format shown in box 1.

Box 1. **Suggested format for reporting antibody information**

Publications using commercial antibodies should report the supplier and the code number. Publications using academic antibodies should report the source laboratory and relevant reference. We recommend the following format;

“The following antibodies were used, Mouse anti-protein A monoclonal antibody (Company E, catalogue number #1000) was used for ELISA with human cells as validated in (figure X or reference Y or validation profile Z) and western blotting in mouse tissue as validated in (figure X or reference Y or validation profile Z).

This format is meant as a guide and could be adapted as required; for example details of batch number, dilution or epitope could be added where particularly important. This information could also be usefully presented in a table if allowed by the journal. Adoption of these reporting guidelines will not eliminate researchers’ frustrations with antibodies, but should help improve experimental reproducibility and scientists’ productivity, something we all seek. An additional benefit for authors who include this information is that well annotated publications are easier for antibody companies and antibody search engines to highlight in their databases. This inclusion is likely to increase the number of researchers who access their work and so potentially the impact of the study.
