# Supplementary material for: Reporting research antibody use: how to increase experimental reproducibility
Source: F1000Res. 2013 Aug 23;2:153. Originally published 2013 Jul 10. [Version 2] doi: 10.12688/f1000research.2-153.v2 (PMC3829129; doi:10.12688/f1000research.2-153.v2)
Supplement: Peer review history

# Peer review: Referee response for version 2

**Published:** 2013-10-01

**Authors:** David Soll

**Affiliations:** 1 University of Iowa, Iowa City, IA, USA

I am happy with the changes and I find the paper acceptable.

I have read this submission. I believe that I have an appropriate level of expertise to confirm that it is of an acceptable scientific standard.

# Peer review: Referee response for version 2

**Published:** 2013-09-09

**Authors:** Simon Glerup

**Affiliations:** 1 Department of Biomedicine, Aarhus University, Aarhus, Denmark

No further revision required.

I have read this submission. I believe that I have an appropriate level of expertise to confirm that it is of an acceptable scientific standard.

# Peer review: Referee response for version 1

**Published:** 2013-08-01

**Authors:** Simon Glerup

**Affiliations:** 1 Department of Biomedicine, Aarhus University, Aarhus, Denmark

This commentary is much needed in the field of life science. It is well written and concise. Andrew Chalmer’s group has contributed significantly to the use of research antibodies by creating CiteAb. When operating the CiteAb search engine, I imagine that they constantly run into problems with publications with poorly described use of research antibodies.

I have two minor suggestions:

1. In the Antibody Validation paragraph, a statement could be included in the methods section of a paper regarding if, where and under what name antibody validation information or reviews has been posted in publically available databases. This would increase the value and transparency of these databases.

1. Unlike the previous reviewer, I think it is fine to mention CiteAb in the paper. After all, even Nature Publishing Group is a highly commercial enterprise. However, I suggest that a table could be included listing the relevant databases including CiteAb, pAbmAbs, Biobrea, Antibodypedia, 1degreebio, Antibody-Advizer etc. In this regard, I regret that the Checklist from Nature Publishing Group only refers to sites in which they have a commercial interest (1degreebio and Antibodypedia). I hope that other publishing groups are not tempted to do the same.

I have read this submission. I believe that I have an appropriate level of expertise to confirm that it is of an acceptable scientific standard.

# Response

**Published:** 2013-09-03

**Authors:** Andrew Chalmers

**Affiliations:** University of Bath, UK

We thank Professor Glerup for his helpful comments and share his concern about the Nature Publishing Group guidelines. We explain our response to each one in turn below.

1. The fact that if no previous validation has occurred then it should be carried out and reported and/or submitted to a public database has been made clearer.  

1. This is a good point and we agree it is important to give an overview of available databases to allow readers to choose the most appropriate. For this reason we were careful to mention a range in our first version. However, we feel it would not be appropriate for us to compile a table given our clear affiliation to one database, instead we provide a link to the most complete list of databases we are aware of.

# Peer review: Referee response for version 1

**Published:** 2013-07-24

**Authors:** John Colyer

**Affiliations:** 1 University of Leeds, Leeds, UK

The title and abstract are clear and appropriate. The article is timely and written clearly and accessibly.

- It could be improved further by providing references for papers that are examples “of good practice and detailed reporting”, which might serve as a template for others.

- The process of antibody validation is worthy of more extensive discussion, as the research community needs to develop a clear understanding of the most appropriate tests to be performed in each experimental system, and standards which should be attained for acceptance of the status of “validated”.  This data should be provided in supplementary data, or by reference to previous supplementary data if the same reagents are used in a new study.

- The importance of batch number is made, but could be emphasized more.

- Finally, the critical role of peer-reviewers in evaluating and enforcing these standards is key. Some discussion of this would enhance the manuscript.

I have read this submission. I believe that I have an appropriate level of expertise to confirm that it is of an acceptable scientific standard.

# Response

**Published:** 2013-09-03

**Authors:** Andrew Chalmers

**Affiliations:** University of Bath, UK

We thank Professor Colyer for his positive and helpful comments and explain our response to each one in turn below.

- A good idea, we have now added an example reference that illustrates good reporting practice (Antibody information is routinely omitted from publications’ section). Journals which already encourage good practice have also been highlighted (‘Change will require help from journals and reviewers’).
** **

- We completely agree and have increased the amount we cover on this topic, but not attempted a full review as we feel such a complex topic is beyond the scope of this comment article. We have added some additional citations for readers who require more information (Antibody Validation section).

- The fact that if no previous validation has occurred then it should be carried out and reported and/or submitted to a public database has been made clearer. The fact that previous validation can be cited has also been spelled out (Antibody Validation section).

- Additional emphasis has been added regarding the problem of batch to batch variability (‘Key details for reporting antibody experiments’ section).

- This has been added to the ‘Change will require help from journals and reviewers’ section.

# Peer review: Referee response for version 1

**Published:** 2013-07-16

**Authors:** David Soll

**Affiliations:** 1 University of Iowa, Iowa City, IA, USA

This commentary is timely and well written, but it could be shortened or tightened up a bit for the purpose of conciseness.  It also should include a few points noted in this review.  The major point is the problem that lack of information in publications involving research antibodies affects assessment and future use.  The discussion could be more efficient in stating that if methods were reported in a previous referenced article, then referencing that article in a new publication is sufficient, unless there are nuances (i.e., new uses of the antibody).  It should also be made clear that such information be mandatory when an antibody is used in a particular way for the first time.  

There are also a few things the author may want to include:

1. Many antibodies work on a particular protein in a particular cell type without knowledge of the protein domain(s) found.  In spite of that they may be of value, so you don’t have to identify the sequence molecule. 

1. Some antibodies identify native conformation and therefore are not on a peptide sequence per se.  Such antibodies are not unusually performed on denatured proteins in western blots, but may work in nature gels. 

1. Some antibodies have not been fully characterized beyond reference to the data sheet provided by the company or source if necessary. 

1.  If the authors of a paper refer to the company, and catalog name of the antibody, prior characterization can access.

1. Antibody validation should go in the supplementary data to a paper.

1. Do not cite CiteAb in your paper - it sounds like an ad. 

But all in all, this is a reasonable commentary.  It reinforces what many already are advocating. The title and abstract were fine.

I have read this submission. I believe that I have an appropriate level of expertise to confirm that it is of an acceptable scientific standard, however I have significant reservations, as outlined above.

# Response

**Published:** 2013-09-03

**Authors:** Andrew Chalmers

**Affiliations:** University of Bath, UK

We thank Professor Soll for his positive review and helpful comments. We have now addressed them and explain our response to each one in turn below: 

*‘The discussion could be more efficient in stating that if methods were reported in a previous referenced article....’*

The fact that previous validation can be cited has now been spelled out more clearly (Antibody Validation section). 

*‘...information be mandatory when an antibody is used in a particular way for the first time’*

The fact that if no previous validation has occurred then validation should be carried out and reported and/or submitted to a public database has been made clearer (Antibody Validation section). These are two key points and we appreciate the fact you raised them.

Things we have now included to respond to the numbered points raised.

1. More discussion of the importance of knowing the antigen for an antibody has been added, in particular raising the point that for some antibodies the antigen is not known, for example when they are raised to a complex cell or tissue lysate (key details for reporting antibody experiments section).  

1. This comment is relevant to the experimental validation of antibodies, we have increased the amount we cover on this topic but not attempted a full review as we feel such a complex topic is beyond the scope of this comment article. We have added some addition citations for readers who require more information (Antibody Validation section).

1. We have made it clearer when validation should be carried out and how it should be reported if no previous validation has taken place (Antibody Validation section).

1. We have now repeated the importance of including catalogue numbers in the antibody validation section.  

1. This is now made clear (Antibody Validation section).  

1. We think giving examples of available antibody databases will be useful to readers and were careful to mention more than one database, we have now added a link to a more extensive list. We have also removed the second reference to CiteAb which was in the final section.
